# Physiological responses to hypoxia are constrained by environmental temperature in heterothermic tenrecs

**DOI:** 10.1242/jeb.245324

**Published:** 2023-03-29

**Authors:** Maiah E. M. Devereaux, Claudia Silva Rubio, Frank van Breukelen, Matthew E. Pamenter

**Affiliations:** ^1^ Department of Biology, University of Ottawa, Ottawa, ON, Canada, K1N 9A7; ^2^ School of Life Sciences, University of Nevada, Las Vegas, Las Vegas, NV 89154, USA; ^3^ University of Ottawa Brain and Mind Research Institute, Ottawa, ON, Canada, K1H 8M5

**Keywords:** Hypercapnia, Hypoxic ventilatory response, Hypoxic metabolic response, Thermogenesis, Metabolic rate depression

## Abstract

Malagasy tenrecs are placental hibernating mammals that seal the entrances to their burrows and hibernate either singly or in groups for 8–9 months, which is likely to create a hypoxic and hypercapnic burrow environment. Therefore, we hypothesized that tenrecs are tolerant to environmental hypoxia and hypercapnia. Many hypoxia- and hypercapnia-tolerant fossorial mammals respond to hypoxia by decreasing metabolic rate and thermogenesis, and have blunted ventilatory responses to both environmental hypoxia and hypercapnia. However, tenrecs exhibit extreme metabolic and thermoregulatory plasticity, which exceeds that of most heterothermic mammals and approaches that of ectothermic reptiles. Thus, we predicted that tenrecs would have abnormal physiological responses to hypoxia and hypercapnia relative to other fossorial mammals. To test this, we exposed common tenrecs (*Tenrec ecaudatus*) to moderate and severe hypoxia (9 and 4% O_2_) or hypercapnia (5 and 10% CO_2_) in either 28 or 16°C while non-invasively measuring metabolic rate, thermogenesis and ventilation. We found that tenrecs exhibit robust metabolic decreases in both hypoxia and hypercapnia. Furthermore, tenrecs have blunted ventilatory responses to both hypoxia and hypercapnia, and these responses are highly temperature sensitive such that they are reduced or absent in 16°C. Thermoregulation was highly variable in 16°C but constrained in 28°C across all treatment conditions and was not impacted by hypoxia or hypercapnia, unlike in other heterothermic mammals. Taken together, our results indicate that physiological responses to hypoxia and hypercapnia in tenrecs are highly dependent on environmental temperature and differ from those of other mammalian heterotherms.

## Teaser

**Highlighted Article:** The metabolic and thermoregulatory responses of Malagasy tenrecs to acute hypoxia and hypercapnia approaches that of ectothermic reptiles.

## INTRODUCTION

Fossorial species may encounter hypoxia and hypercapnia on an intermittent or sustained basis owing to group respiration, poor ventilation and air circulation, and/or limited gas diffusion through the soil (Lacey et al., 2000; Shams et al., 2005). Indeed, burrow measurements from various fossorial and subterranean species span ambient O_2_ concentrations of 6–20.5% and CO_2_ concentrations of 4–9.5% for active (non-hibernating) animals (Kennerly, 1964; Kuhnen, 1986; Maclean, 1981; Roper et al., 2001; Schaefer and Sadleir, 1979). Furthermore, in burrows sealed for hibernation, gas concentrations can reach as low as 4% O_2_ and as high as 13.5% CO_2_ (Kuhnen, 1986; Williams and Rausch, 1973). Such extreme hypoxic environments can exert a strong evolutionary pressure (Pamenter et al., 2020), and so it is not surprising that animals inhabiting these environments have acquired complex physiological and molecular adaptations to these otherwise intolerable conditions (Boggs et al., 1984; Dzal et al., 2015; Lui et al., 2015).

Hypoxia is primarily an energetic challenge and compromises aerobic ATP production. As such, most hypoxia-tolerant fossorial mammals reduce metabolic demand to match the reduced capacity for aerobic energy production in hypoxia (Buck and Pamenter, 2006; Dzal et al., 2015; Frappell et al., 1992; Guppy and Withers, 1999; Hochachka et al., 1996). This is termed the hypoxic metabolic response (HMR) and a major component of the HMR is downregulation of thermogenesis and body temperature (*T*_b_) setpoint (Tattersall and Milsom, 2009; Wood, 1991). Conversely, hypercapnia impedes the ability to expel CO_2_ via exhalation, resulting in an accumulation of CO_2_ in the blood and tissues, and thus respiratory acidosis (Birchard et al., 1984). The metabolic response to hypercapnia varies across mammals and has no discernible pattern amongst fossorial species (Barros et al., 1998; Devereaux et al., 2021; Marques et al., 2015; Mortola and Lanthier, 1996; Sachdeva and Jennings, 1994; Zhang and Pamenter, 2019). Beyond metabolic adaptations, the primary reflex response to either hypoxia or hypercapnia is to increase ventilation, which is referred to as the hypoxic ventilatory response (HVR) and the hypercapnic ventilatory response (HCVR), respectively (Frappell et al., 1992; Iturriaga et al., 2016; Pamenter and Powell, 2016; Powell et al., 1998). In fossorial mammals, the HVR and HCVR are generally smaller than those of non-fossorial mammals and manifest at deeper levels of hypoxia and hypercapnia. Because of this, fossorial mammals are said to have a ‘blunted’ HVR and/or HCVR. For example, in fossorial mammals, the HVR threshold ranges from 5 to 12.5% O_2_, whereas in non-fossorial mammals, ventilation increases in ∼15–16% O_2_ (Arieli et al., 1977; Devereaux and Pamenter, 2020; Frappell et al., 1992; Guppy and Withers, 1999; Ivy et al., 2020; Tomasco et al., 2010). Similarly, the HCVR in fossorial mammals is initiated in 4.5–8% CO_2_, whereas the threshold for non-fossorial mammals is lower, manifesting in as little as 1% CO_2_ (Arieli and Ar, 1979; Barros et al., 2004; Boggs et al., 1998; Devereaux and Pamenter, 2020; Frappell et al., 1994; Ivy et al., 2020; Tomasco et al., 2010).

Tenrecs are placental mammals from Madagascar, and have many plesiomorphic traits (Eisenberg, 1981; Treat et al., 2018). The common tenrec (*Tenrec ecaudatus*) housed at ambient temperatures (*T*_a_) of 12 to 28°C may be torpid with *T*_b_ approaching *T*_a_ (Treat et al., 2018). Furthermore, tenrecs are continuously torpid during the hibernation season with no periodic euthermic arousals, which punctuate torpor bouts of all other known small hibernators. More interestingly, active tenrecs may have a *T*_b_ similar to that of hibernating animals. For example, an animal maintained at 12°C may be fully active and even swim with a *T*_b_ of ∼13°C (Treat et al., 2018). However, active *T*_b_ is variable with temperatures that frequently wax and wane to as high as ∼34°C depending on *T*_a_ (Treat et al., 2018). Concomitantly, resting oxygen consumption rates (*V̇*_O_2__) of active animals vary by as much as 25-fold when active animals are maintained at 12°C, such that the *V̇*_O_2__ of metabolically low active tenrecs resembles that of hibernating tenrecs (Treat et al., 2018). Aside from this large variation in aerobic metabolism between active tenrecs, there are prominent physiological disconnects within individual tenrecs, such that relationships between heart rate, *V̇*_O_2__ and *T*_b_ are unpredictable.

The common tenrec is defined as terrestrial to semi-fossorial (Everson et al., 2016; Poux et al., 2008; Stankowich and Stensrud, 2019). Regardless of season, whether active or hibernating, these tenrecs construct burrows up to 1.5 m deep (Eisenberg and Gould, 1970; Rand, 1935). Common tenrecs completely seal burrow entrances for the 8- to 9-month hibernation season, and video evidence suggests hibernating tenrecs use communal burrows (e.g. 13 torpid tenrecs were removed from a burrow; Treat et al., 2018); sealed burrows with 13 tenrecs are likely to become hypoxic and hypercapnic. Thus, an obvious question is what is the hypoxia and hypercapnia tolerance of tenrecs? Given the strong pressures that environmental hypoxia and hypercapnia exert on metabolism, thermoregulation and ventilation, and the seemingly unique disconnects between these physiological components in this species, we measured metabolic, thermoregulatory and ventilatory characteristics in active tenrecs at 16 and 28°C during hypoxia and hypercapnia. Owing to the remarkable number of plesiomorphic traits and extreme metabolic and thermoregulatory plasticity of tenrecs, we posited that they may be more reminiscent of an early placental mammalian ancestor than any other extant mammal (O'Leary et al., 2013). In other words, we suggest here that perhaps we can understand the hypoxic or hypercapnic responses of an early mammal by studying tenrecs.

## MATERIALS AND METHODS

### Animals

Tenrecs [*Tenrec ecaudatus* (Schreber 1778); ∼2 years old] were individually housed in an environmentally controlled room with 20.95% O_2_, 0.04% CO_2_, 15% humidity and a 12 h:12 h light:dark light cycle. Animals were fed Mazuri Insectivore diet (St Paul, MN, USA) supplemented with Purina Puppy Chow (St Louis, MO, USA). Animals were fed at the same time each day and were not fasted prior to experimental trials because the variability in *V̇*_O_2__ of active tenrecs is not affected by feeding status (Crompton et al., 1978; Treat et al., 2018). All experimental procedures were approved by the University of Ottawa's Animal Care Committee (protocol 2535) in accordance with the Animals for Research Act and the Canadian Council on Animal Care and the University of Nevada, Las Vegas Institutional Animal Care and Use Committee (protocol IACUC-01176). Wild-caught common tenrecs were imported to the University of Nevada under federal and state permit from Mauritius in June 2014.

### Experimental design

Seven male tenrecs weighing 1001±22 g (mean±s.e.m.) were subjected to three different experimental protocols: (1) normoxia, (2) acute graded hypoxia and (3) acute graded hypercapnia. In the first protocol, animals were held at 20.95% O_2_/0.04% CO_2_ for 2 h. In the second protocol, animals were held in normoxia to establish a baseline, and were then subjected to two exposures of progressively deeper hypoxia, followed by a reoxygenation period (i.e. 21, 9, 4 and 21% O_2_; balance N_2_, 30 min each). In the third protocol, animals were held in normocapnia to establish a baseline, and were then subjected to two exposures of progressively deeper hypercapnia, followed by a period of normocapnia (i.e. 0, 5, 10 and 0% CO_2_; 21% O_2_, balance N_2_, 30 min each). All experiments were performed at both 16 and 28°C. Previous observations (F.v.B., unpublished observations) suggest that 2–3 days are required for tenrecs to acclimate to a new environmental temperature; therefore, animals were allowed 4 days at each temperature prior to experimentation. Experimental conditions were not randomized, but tenrecs were given >1 day between gas conditions to eliminate pre-conditioning effects.

### Whole-body plethysmography and respirometry

Animals were individually placed, unrestrained, in a 4.0 liter Plexiglass chamber. Attached to the experimental chamber was an identical second chamber that acted as a reference. The animal chamber was sealed and continuously ventilated with gas mixtures set to the desired fractional gas composition by calibrated rotameters (Krohne, Duisburg, Germany). Inflowing gas was set at a flow rate of 1100 ml min^−1^, as determined using a Field Metabolic System (FMS; Sable Systems International, Las Vegas, NV, USA), which also measured humidity, O_2_ concentration and CO_2_ concentration of the expired air. After humidity was measured and before entering the cells of the CO_2_ and O_2_ analyzers, the excurrent gas was passed through a desiccant medium (Drierite, W.A. Hammond Drierite Co. Ltd, Xenia, OH, USA). Before each trial, the O_2_ and CO_2_ analyzers were calibrated using 100% N_2_ and compressed air (20.95% O_2_, 0.04% CO_2_). For 5 min at the end of each 30-min period, incurrent gas concentrations were measured by bypassing the experimental chamber and diverting air flow directly to the FMS. In the hypoxia or hypercapnia trials, gas concentrations were then changed to the preceding O_2_ concentration before returning airflow to the experimental chamber.

Animal inspiration caused pressure fluctuations owing to changes in humidity and warmth between inspired and expired air. These fluctuations were compared with the pressure of the identical reference chamber. Continuous monitoring by a differential pressure transducer (DP103-18, Validyne, Northridge, CA, USA) connected between the two chambers amplified this signal, thereby allowing us to detect and measure breaths. Before each trial, the transducer was calibrated by injecting five known volumes of air (0.1, 0.2, 0.3, 0.4 and 0.5 ml) 10 times into the experimental chamber. *T*_b_ was recorded non-invasively using previously implanted subcutaneous Tempo Discs (BlueMaestro, London, UK) calibrated to sample every 10 min. Chamber temperature was recorded at every inflow period using a custom designed thermocouple.

### Data collection and analysis

Metabolic data were collected and analyzed using Expedata (Sable Systems International). O_2_ and CO_2_ concentrations and pressure deflections were sampled at 100 Hz. The final 10 min of stable activity and breathing at the end of each 30-min period was analyzed to calculate metabolic and ventilatory parameters. Periods where the animal was exercising were excluded from this measurement. To estimate metabolic rate, the average O_2_ and CO_2_ concentrations from this 10-min period were measured. Incurrent gas concentrations and relative humidity (%) were taken from a 30-s period of stable concentration during the inflow measurement.

Using these measurements, the rate of oxygen consumption (*V̇*_O_2__) was calculated using eqn 10.6 in Lighton (2008):
(1)

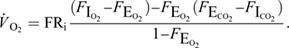
The rate of CO_2_ production (*V̇*_CO_2__) was calculated using eqn 10.7 from the same source:
(2)

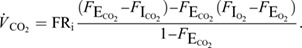


In both equations, FR_i_ is the incurrent flow rate (ml min^−1^), *F*i_O_2__ and *F*i_CO_2__ are the fractional concentrations of incurrent O_2_ and CO_2_ of dry gas, respectively, and *F*e_O_2__ and *F*e_CO_2__ are the fractional concentrations of excurrent O_2_ and CO_2_ from the experimental chamber, respectively. The equations were modified to include body mass (ml min^−1^ kg^−1^). The respiratory exchange ratio (RER) was calculated as a ratio of *V̇*_CO_2__/*V̇*_O_2__. The % of O_2_ extracted (*E*_O_2__) was calculated as: [(*F*i_O_2__–*F*e_O_2__)/*F*i_O_2__]×100.

Ventilatory data were collected using LabChart software (ADInstruments, Colorado Springs, CO, USA) and analyzed in PowerLab (ADInstruments). To calculate tidal volume (*V*_T_) and respiratory frequency (*f*_R_), five breath sets were selected from within the same 10-min period used for metabolic rate calculations, with each set consisting of a minimum of 10 consecutive and clearly defined breaths. Each pressure oscillation was counted as one breath. The Drorbaugh and Fenn equation:
(3)

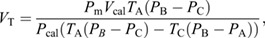
was used to calculate *V*_T_ (Drorbaugh and Fenn, 1955). *P*_m_ (measured in V) is the pressure deflection of the expired breaths. The average cyclic height of deflections was taken from each breath set, representing the average total pressure deflection of a breath, *P*_m_. *P*_cal_ (V) and *V*_cal_ (µl) are the pressure deflection and volume of a known calibrated volume, respectively. The average cyclic deflection height of each calibration set was plotted against the injected volume to create a linear relationship. The point on this line representing 0.2 ml was chosen as *P*_cal_ and *V*_cal_. *T*_A_ and *T*_C_ are the animal body and chamber temperature (K), respectively, each recorded at the end of the 10-min period. *P*_B_ is the barometric pressure in the laboratory (mm Hg) as measured by the O_2_ analyzer. *P*_B_ in Las Vegas, NV, USA, ranged from 93 to 95 kPa during these experiments. *P*_A_ is the vapour pressure of water at the animal's body temperature (mm Hg) and *P*_C_ is the partial pressure of water vapour (mm Hg) in the incurrent gas stream. *P*_A_ was calculated using relative humidity (%) of excurrent air, animal temperature (°C) and barometric pressure (kPa). *P*_C_ used relative humidity (%) of incurrent air, chamber temperature (°C) and barometric pressure (kPa). Minute ventilation (*V̇*_E_) was calculated as the product of *f*_R_ and *V*_T_. The air convection requirements of O_2_ and CO_2_ (ACR_O_2__ and ACR_CO_2__) were calculated as the quotient of *V̇*_E_ and *V̇*_O_2__ or *V̇*_CO_2__, respectively.

### Statistical analysis

Statistical analysis was performed using commercial software (Prism v.9.2.0, GraphPad Software Inc., San Diego, CA, USA). All values are presented as means±s.e.m., where *P*<0.05 was the threshold for significance. Owing to the wide variance, sphericity was not assumed. This was corrected for using a Geisser–Greenhouse correction. Statistical significance was evaluated using three-way repeated measures (RM) ANOVA to test for interactions between two independent variables: normoxia versus hypoxia/hypercapnia and temperature (16 versus 28°C). A Tukey's multiple comparisons test was performed on each dependent variable to determine significance.

## RESULTS

### Tenrec metabolism is highly plastic but is constrained by temperature and hypoxia

**Fig. 1.**
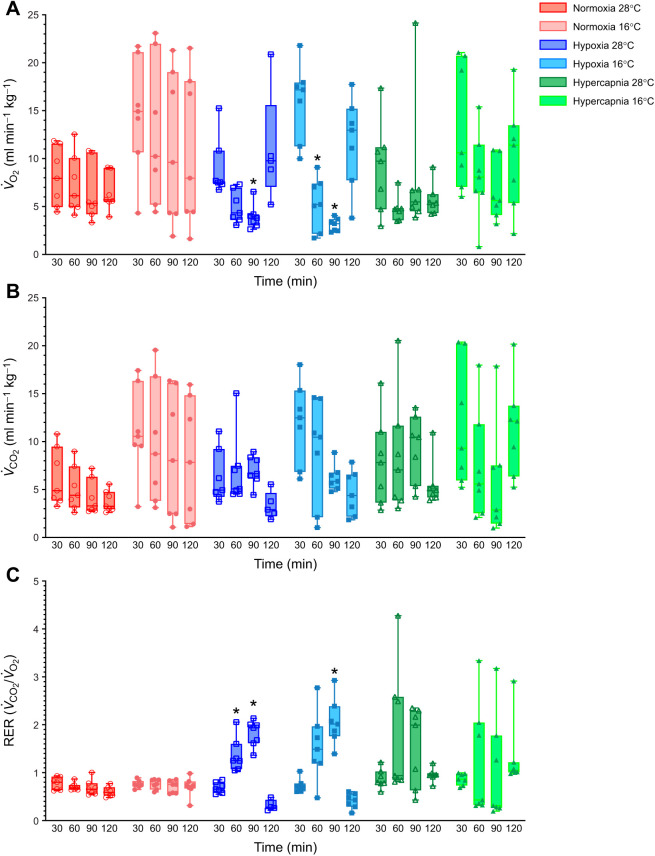
**Tenrecs have a robust hypoxic metabolic response, which is enhanced in cold temperatures.** Summaries of (A) O_2_ consumption rate (*V̇*_O_2__), (B) CO_2_ production rate (*V̇*_CO_2__) and (C) respiratory exchange ratio (RER) from 7 male tenrecs exposed to normoxia (2 h of 21% O_2_; red circles), hypoxia (30 min normoxia, 30 min each of 9 and then 4% O_2_, and 30 min recovery in normoxia; blue squares) or hypercapnia (30 min normocapnia, 30 min each of 5 and then 10% CO_2_, and 30 min recovery in normocapnia; green triangles) in 28°C (open symbols, dark shades) and 16°C (closed symbols, light shades). Data are presented as box and whisker plots, which include the median line, upper and lower quartiles, and data range. Asterisks (*) indicate significant difference from normoxic/normocapnic controls in the same experiment protocol (three-way ANOVA with Tukey's *post hoc* tests, *P<*0.05).

We first evaluated metabolic changes in tenrecs exposed to normoxia, hypoxia or hypercapnia at two experimental temperatures. A three-way ANOVA indicated that there was no significant effect of experimental temperature on *V̇*_O_2__ (*F*_1,12_=1.392, *P*=0.2609; Fig. 1A) or *V̇*_CO_2__ (*F*_1,12_=0.01156, *P*=0.9161; Fig. 1B). However, in animals acclimated to 16°C and breathing normoxia, metabolic rate (as measured indirectly by both *V̇*_O_2__ and *V̇*_CO_2__) was highly variable, with individual awake tenrecs having *V̇*_O_2__ values varying ∼10-fold (from 2 to 23 ml min^−1^ kg^−1^) and *V̇*_CO_2__ values varying ∼20-fold (from 1 to 20 ml min^−1^ kg^−1^; Fig. 1A,B, light red bars). In 28°C experiments, these ranges were constrained at both the upper and lower bounds to a reduced range of approximately one-third those of animals acclimated to 16°C (dark red bars).

In hypoxia, metabolic rates were constrained in both 16 and 28°C and animals exhibited a robust reduction in *V̇*_O_2__ relative to normoxic controls (*F*_3,34_=5.591, *P*=0.0032). The degree of both the reduction in the variability across the metabolic rates of individuals and the overall mean metabolic rate depression increased with more severe hypoxia (*F*_1.389,16.67_=29.82, *P*<0.0001). Similarly, hypoxia had an overall significant effect on *V̇*_CO_2__ relative to normoxic controls (*F*_3,34_=3.80, *P*=0.0188; Fig. 1, blue bars) and with deeper hypoxia (*F*_1.294,15.53_=16.75, *P*=0.0005), although *V̇*_CO_2__ did not decrease significantly at any individual time point. However, the range of *V̇*_CO_2__ measurements became more constrained with deeper hypoxia in 16°C relative to normoxic experiments in the same temperature. *V̇*_O_2__ did not change significantly in hypercapnia (*F*_3,36_=1.500, *P*=0.2311), but became constrained in hypercapnia in 28°C but not in 16°C, whereas *V̇*_CO_2__ was not significantly impacted by hypercapnia in either temperature (*F*_1,12_=3.312, *P*=0.0938).

Comparing the rates of O_2_ consumption and CO_2_ production provides an indirect measure of metabolic fuel usage, such that values near 0.7 indicate primarily lipid metabolism, values near 1.0 indicate primarily carbohydrate metabolism, and variables outside this range indicate animals that are not in steady state or that utilize abnormal metabolic adaptations (Lau et al., 2017; Simonson and DeFronzo, 1990). Analysis of these RERs indicated that tenrecs primarily metabolized lipids in normoxia, and that this metabolic fuel preference was not impacted by temperature (Fig. 1C).

In hypoxic experiments, RERs increased in both experimental temperatures (*F*_2.077,24.92_=55.78, *P*<0.0001), and this change was primarily driven by a disconnect between *V̇*_O_2__ and *V̇*_CO_2__, which decreased and remained unchanged, respectively. In hypercapnic experiments, RERs did not change significantly but became highly variable (*F*_1.899,22.79_=1.580, *P*=0.2280).

### Tenrecs have a blunted HVR and HCVR that is further reduced at the lower temperature

**Fig. 2.**
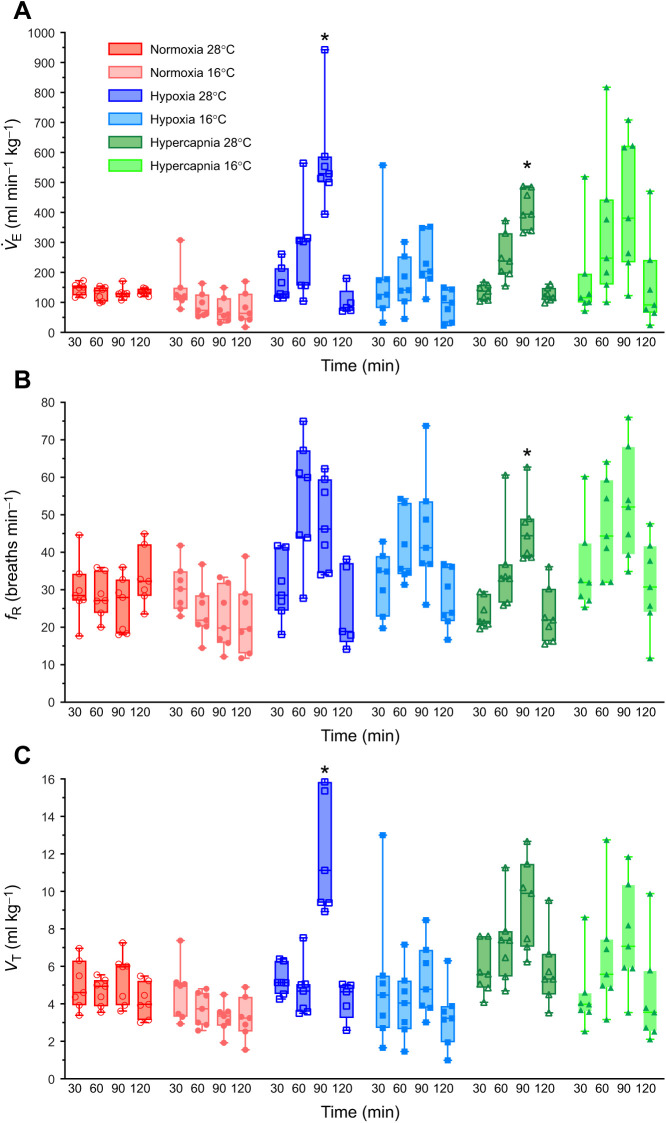
**Ventilatory responses of tenrecs to hypoxia and hypercapnia are blunted and constrained by ambient temperature.** Summaries of (A) minute ventilation (*V̇*_E_), (B) breathing frequency (*f*_R_) and (C) tidal volume (*V*_T_) from 7 male tenrecs exposed to normoxia (2 h of 21% O_2_; red circles), hypoxia (30 min normoxia, 30 min each of 9 and then 4% O_2_, and 30 min recovery in normoxia; blue squares) or hypercapnia (30 min normocapnia, 30 min each of 5 and then 10% CO_2_, and 30 min recovery in normocapnia; green triangles) in 28°C (open symbols, dark shades) and 16°C (closed symbols, light shades). Data are presented as box and whisker plots. Asterisks (*) indicate significant difference from normoxic/normocapnic controls in the same experiment protocol (three-way ANOVA with Tukey's *post hoc* tests, *P<*0.05).

Next, we measured changes in *V̇*_E_ and its component parameters (*f*_R_ and *V*_T_) to evaluate the HVR and HCVR. *V̇*_E_, *f*_R_ and *V*_T_ were relatively stable in tenrecs held in normoxia, and there was a significant effect of temperature on all three parameters (*F*_1,12_=18.38, *P*=0.0011 for *V̇*_E_, *F*_1,12_=45.13, *P*<0.0001 for *f*_R_, and *F*_1,12_=8.305, *P*=0.0138 for *V*_T_; Fig. 2).

There was also a significant effect of hypoxia on *V̇*_E_ (*F*_3,34_=12.14, *P*<0.0001), such that *V̇*_E_ increased ∼4-fold in animals breathing 4% O_2_ in 28°C experiments (*P*=0.0046). Conversely, *V̇*_E_ did not change in animals breathing 9% O_2_ in 28°C experiments (*P*=0.4642). The increase in *V̇*_E_ in 4% O_2_ was primarily driven by an increase in *V*_T_ (*F*_1,34_=13.38, *P*=0.0009 overall interaction; *P*=0.0353 for 4% O_2_) and a non-significant increase in *f*_R_ (*F*_1,34_=1.816, *P*=0.1867 overall interaction; *P*=0.3409 for 4% O_2_), whereas neither parameter increased significantly in 9% O_2_ (*P*=0.9920 and 0.0810 for *V*_T_ and *f*_R_, respectively). In 16°C experiments, the impact of hypoxia was abrogated and no ventilatory parameter changed with hypoxia at this temperature.

There was also a significant effect of hypercapnia on *V̇*_E_ (*F*_1.258,15.10_=18.89, *P*=0.0003), *f*_R_ (*F*_1.751,21.01_=8.437, *P*=0.0028) and *V*_T_ (*F*_1.586,19.03_=12.64, *P*=0.0006). In animals acclimated to 28°C, *V̇*_E_ and *f*_R_ increased in 10% CO_2_ (*P*=0.0013 and 0.0421), whereas *V*_T_ approached significance (*P*=0.0657). Conversely, none of these variables changed in 5% CO_2_ in animals held in 28°C, or in animals breathing hypercapnia in 16°C. The lack of significance in the 16°C hypercapnia dataset was primarily due to the wide variability in ventilatory responses to hypercapnia, as the means of *V̇*_E_, *f*_R_ and *V*_T_ all increased non-significantly with progressive hypercapnia, but individual animal *V̇*_E_ responses (for example) varied by as much as 8-fold in these experiments.

### Thermogenesis is constrained by ambient temperature but is not impacted by hypoxia or hypercapnia

**Fig. 3.**
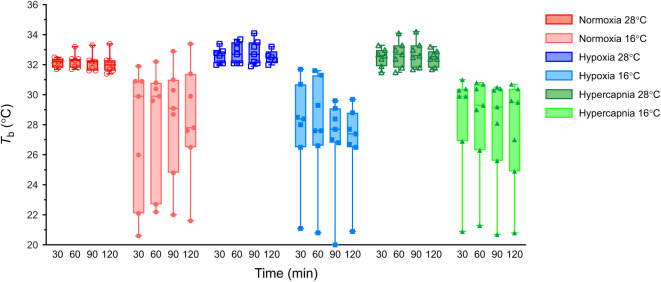
**Body temperature (*T*_b_) is constrained by ambient temperature but not hypoxia or hypercapnia.** Summary of *T*_b_ from 7 male tenrecs exposed to normoxia (2 h of 21% O_2_; red circles), hypoxia (30 min normoxia, 30 min each of 9 and then 4% O_2_, and 30 min recovery in normoxia; blue squares) or hypercapnia (30 min normocapnia, 30 min each of 5 and then 10% CO_2_, and 30 min recovery in normocapnia; green triangles) in 28°C (open symbols, dark shades) and 16°C (closed symbols, light shades). Data are presented as box and whisker plots.

Thermoregulation is an energetically demanding process in small mammals and many such species reduce *T*_b_ to facilitate metabolic savings in hypoxia (Barros et al., 2001; Wood and Gonzales, 1996; Wood and Stabenau, 1998). Therefore, we also measured *T*_b_ to gain insight into the regulation of thermogenesis in hypoxia and hypercapnia. Overall, temperature did not have a significant effect on *T*_b_ (*F*_1,12_=0.0413, *P*=0.8424). However, tenrecs acclimated to 28°C maintained *T*_b_ in a relatively tight range of ∼32–33°C in normoxia, hypoxia and hypercapnia (Fig. 3). Conversely, in animals acclimated to 16°C, *T*_b_ varied widely, between ∼20 and 32°C. *T*_b_ was not impacted by changes in gas concentrations in either temperature (*F*_1.122,13.46_=0.6456, *P*=0.4527 for hypoxia and *F*_1.071,12.85_=0.6614, *P*=0.4411 for hypercapnia).

### Tenrecs hyperventilate in hypoxia and hypercapnia in 28°C but not in 16°C

**Fig. 4.**
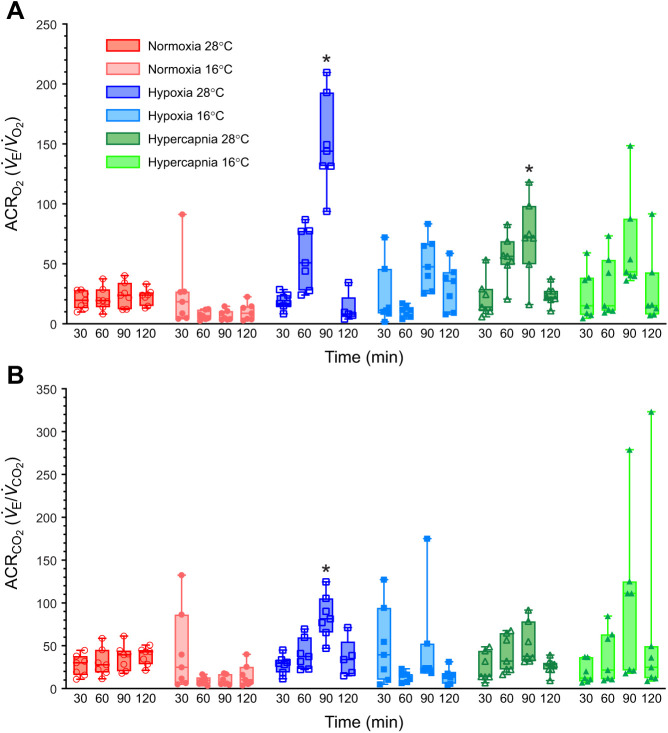
**Tenrecs hyperventilate in hypoxia and hypercapnia in warm but not cold temperatures.** Summaries of the air convection requirement of (A) O_2_ (ACR_O_2__) and (B) CO_2_ (ACR_CO_2__) from 7 male tenrecs exposed to normoxia (2 h of 21% O_2_; red circles), hypoxia (30 min normoxia, 30 min each of 9 and then 4% O_2_, and 30 min recovery in normoxia; blue squares) or hypercapnia (30 min normocapnia, 30 min each of 5 and then 10% CO_2_, and 30 min recovery in normocapnia; green triangles) in 28°C (open symbols, dark shades) and 16°C (closed symbols, light shades). Data are presented as box and whisker plots. Asterisks (*) indicate significant difference from normoxic/normocapnic controls in the same experiment protocol (three-way ANOVA with Tukey's *post hoc* tests, *P<*0.05).

ACRs are a useful integrated indicator of hyperventilation as they provide a combined measure of *V̇*_E_ relative to metabolic demand. Therefore, we next calculated ACRs to determine whether tenrecs hyperventilate in hypoxia or hypercapnia relative to their metabolic needs. In normoxia, ACRs were relatively consistent in animals acclimated to both 16 and 28°C (Fig. 4A,B). Conversely, there was a significant effect of hypoxia on both the ACR_O_2__ (*F*_2.114,25.37_=6.267, *P*=0.0055) and ACR_CO_2__ (*F*_2.233,26.79_=33.23, *P*<0.0001). Specifically, ACRs were both elevated in moderate hypoxia (9% O_2_), albeit not to a significant degree, whereas both ACRs became significantly elevated in severe hypoxia in animals acclimated to 28°C (*P*=0.0019 and 0.0046 for ACR_O_2__ and ACR_CO_2__, respectively). Conversely, in animals acclimated to 16°C, neither ACR increased at any level of hypoxia. Finally, the ACR_O_2__ increased with severe hypercapnia in animals acclimated to 28°C (*P*=0.0166), but this change did not occur in similarly treated animals acclimated to 16°C.

### Oxygen extraction increases in severe hypoxia but not hypercapnia

**Fig. 5.**
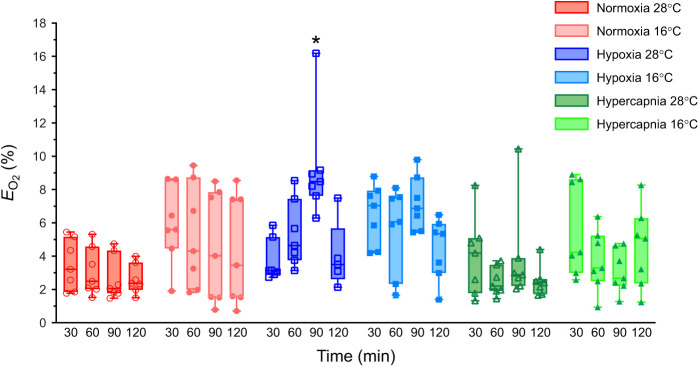
**The percent of O_2_ extracted (*E*_O_2__) increases in hypoxia in a temperature-dependent fashion.** Summary of the *E*_O_2__ from 7 male tenrecs exposed to normoxia (2 h of 21% O_2_; red circles), hypoxia (30 min normoxia, 30 min each of 9 and then 4% O_2_, and 30 min recovery in normoxia; blue squares) or hypercapnia (30 min normocapnia, 30 min each of 5 and then 10% CO_2_, and 30 min recovery in normocapnia; green triangles) in 28°C (open symbols, dark shades) and 16°C (closed symbols, light shades). Data are presented as box and whisker plots. Asterisk (*) indicates a significant difference from normoxic/normocapnic controls in the same experiment protocol (three-way ANOVA with Tukey's *post hoc* tests, *P<*0.05).

Lastly, we measured the percent of O_2_ extracted in each breath (*E*_O_2__), which provides an indirect measure of pulmonary gas exchange and reflects changes in diffusion barriers and/or blood O_2_ carrying and exchange properties (Bing et al., 1949). In this analysis, we found that there was a significant effect of hypoxia on *E*_O_2__ (*F*_1.640,19.68_=9.951, *P*=0.0017), such that this variable increased ∼3-fold in severe hypoxia in animals acclimated to 28°C (*P*=0.0359; Fig. 5). Conversely, *E*_O_2__ was unchanged at other levels of hypoxia, in animals acclimated to 16°C breathing hypoxic gas mixtures, and in animals breathing hypercapnia in either temperature.

## DISCUSSION

We evaluated the hypothesis that common tenrecs, which group-hibernate for 8–9 months in sealed burrows and exhibit remarkable metabolic plasticity, are hypoxia and hypercapnia tolerant. We predicted that, like other semi-fossorial and heterothermic species, tenrecs have a robust metabolic response to hypoxia, blunted ventilatory responses to both hypoxia and hypercapnia, and decreased thermogenesis in hypometabolic conditions. Our results provide mixed support for our hypothesis and predictions, but the major finding of our study is that all physiological characteristics were primarily sensitive to environmental temperature and not O_2_ or CO_2_ levels. Specifically, the physiological phenotype of tenrecs in normoxia and their physiological responses to hypoxia and hypercapnia are constrained and shaped by experimental temperature. Notably, the relationship between these variables and temperature is like that found in heterotherms and even ectotherms, including heterothermic mammals, and ectothermic reptiles, fish and amphibians. It is also notable that animals held in the cold temperature exhibit considerably greater metabolic and thermoregulatory variability relative to animals in the warmer temperature. Furthermore, there is no clear coupling of these variables, which is consistent with a previous study in this species but different from homeothermic and heterothermic mammals in which thermogenesis and metabolic rate are typically directly linked (Treat et al., 2018). Taken together, our results suggest that the extreme metabolic and thermoregulatory plasticity of tenrecs (Treat et al., 2018) is the predominant characteristic that defines and regulates their physiological responses to gaseous environmental challenges.

### Tenrecs have blunted ventilatory responses to hypoxia and hypercapnia and a robust HMR

Consistent with our initial hypothesis and predictions, tenrecs exhibit blunted ventilatory responses to both hypoxia and hypercapnia, and have robust metabolic responses to both conditions. Specifically, in 28°C experiments, tenrec ventilation is not significantly elevated in 9% O_2_ or 5% CO_2_ (although a larger sample size would likely have revealed a change in 5% CO_2_), with ventilation only becoming elevated in the more extreme conditions (4% O_2_ and 10% CO_2_). This blunting of the sensitivity of the HVR and HCVR is consistent with most studies in other hypoxia-tolerant fossorial and semi-fossorial mammals, including solitary and social mole-rats, heterothermic and homeothermic rodents, and species that hibernate in sealed burrows alone and in groups (Arieli and Ar, 1979; Arieli et al., 1977; Barros et al., 2004; Boggs et al., 1998; Devereaux and Pamenter, 2020; Frappell et al., 1992, 1994; Guppy and Withers, 1999; Ivy et al., 2020; Tomasco et al., 2010). Interestingly, the HVR and HCVR were further blunted and largely absent in 16°C experiments (see further discussion below). Also, like studies in most other hypoxia-tolerant fossorial species, tenrecs exhibited a robust HMR and a strong metabolic response to hypercapnia. However, and unlike in all other fossorial mammals studied to date, tenrecs do not exhibit a significant change in *T*_b_ with progressive hypoxia. As a result of the combination of these changes, tenrecs hyperventilate in both conditions, with the ventilatory equivalent increasing nearly 7-fold in severe hypoxia and ∼3-fold in severe hypercapnia. However, these changes are reduced and not significant in moderate hypoxia and hypercapnia and are absent in 16°C experiments. In hypercapnia in 16°C conditions in particular, the significance of change was lost owing to increased variability in the response of tenrec metabolism and ventilation to inhaled CO_2_.

### Physiological responses to hypoxia are temperature sensitive and like those of heterotherms and ectotherms

An intriguing finding of our study is the observation that both the sensitivity and the magnitude of the HVR decrease with decreasing temperature in tenrecs. This result is similar to studies in other small adult heterothermic mammals (measured in the active state), but contrasts to data from homeothermic mammals, in which the magnitude of the HVR is maintained with progressive cooling above the lower critical temperature. Specifically, *V̇*_E_ and *V̇*_O_2__ decrease with decreasing environmental temperature in adult heterotherms, including facultatively heterothermic hamsters (*Mesocricetus aeratus*) and mice (*Mus musculus*), and obligatory heterothermic ground squirrels (*Ictidomys tridecemlineatus*) breathing 7% O_2_, but not in homeothermic adult rats (*Rattus norvegicus*) (Dzal and Milsom, 2019). Conversely, in neonatal animals from the same four species, *f*_R_, *V̇*_E_ and *V̇*_O_2__ decrease steadily with decreasing environmental temperature, although *V*_T_ increases (Dzal and Milsom, 2021). Notably, adult homeothermic animals are able to defend their *T*_b_ to some degree in hypoxia, whereas heterotherms achieve this to a lesser degree. Conversely, neonates (like ectotherms) are unable to defend *T*_b_, such that the difference between *T*_b_ and *T*_a_ is maintained during progressive cooling in hypoxia as both variables decrease in tandem (Dzal and Milsom, 2019, 2021).

It is intriguing to note that decreases in the HVR with colder environmental temperatures also occur in ectotherms. For example, in the anoxia-tolerant pond turtle *Pseudomys scripta elegans*, the magnitude of the HVR decreases and the hypoxic threshold at which the HVR is activated increases (i.e. deeper hypoxia is required to elicit hyperventilation) with progressively cooler environmental temperatures (Jackson, 1973). Notably, these turtles also substantially decrease their metabolic demand in hypoxia/anoxia, which supports minimal hyperventilation in severe hypoxia in the cold. Similar results have been reported from numerous other ectotherms, including reptiles, amphibians and fish. For example, in several species of green lizards (*Lacerta viridis*), geckos (*Tarentola mauretanica*), the green iguana (*Iguana iguana*), toads (*Bufo marinus* and *B. paracnemis*), frogs (*Rana catesbeiana*), alligators and common carp (*Cyprinus carpio*), hypoxia decreases *V̇*_E_ and *V̇*_O_2__, whereas the threshold at which the HVR is initiated and the magnitude of change in the HVR both decrease with decreasing environmental temperature (Branco et al., 1993; Dupre et al., 1989; Gamperl et al., 1999; Giordano and Jackson, 1973; Nielsen, 1962; Porteus et al., 2011; Rocha and Branco, 1998; Stecyk and Farrell, 2002).

### Conclusions

Given the consistent pattern in the relationship between the HVR and environmental temperature in homeotherms and ectotherms, it is interesting to consider that, in Lovegrove's plesiomorphic–apomorphic endothermy model, mammals with a *T*_b_ below 35°C are classified as protoendothermic or basoendothermic (Lovegrove, 2012). This model predicts that early mammals likely had variable and lower *T*_b_ values. Owing to several examples of at least one heterothermic species existing in distinct mammalian orders, it is assumed that heterothermy is a plesiomorphic character and endothermy an apomorphic character (Lovegrove, 2012), but tenrecs may approach ectothermy in their physiological response to hypoxia. Indeed, early work on *V̇*_O_2__ of exercising common and greater hedgehog tenrecs (*T. ecaudatus* and *Setifer setosus*), hedgehogs, opossums and echidnas revealed tenrecs to be more similar to a hypothetical, exercising reptile than to a hypothetical mammal of the same size (Crompton et al., 1978). In other words, while seemingly bizarre, the metabolic and thermoregulatory plasticity seen in common tenrecs may simply be reflective of an ancestral condition.

Thus, the difference between the thermal sensitivity of the HVR in adult tenrecs relative to adult homeothermic mammals may be due to reduced thermogenic capacity and/or a lower drive to maintain thermal homeostasis, approaching that of ectothermic species. Specifically, most adult heterothermic and homeothermic mammals (to some degree) attempt to defend *T*_b_ in the cold in hypoxia, and thus *V̇*_O_2__ increases, requiring increased *V̇*_E_. Conversely, *V̇*_O_2__ in ectotherms is more closely related to passive *Q*_10_ effects, such that metabolic rates increase in warmer temperatures and vice versa. Studies examining the regulation of thermogenic mechanisms in tenrecs exposed to hypoxia in the cold will be important to understand this relationship, particularly because decreased *V̇*_O_2__, *V̇*_E_ and *T*_b_ in mammalian neonates during hypoxia is primarily due to decreasing and eventually turning off non-shivering thermogenesis (Mortola and Lanthier, 1996; Teppema and Dahan, 2010). We also acknowledge that a group of tenrecs may respond differently than an individual tenrec to hypoxia or hypercapnia, and therefore further investigation of physiological responses to hypoxia/hypercapnia in group-housed animals is warranted.
